# Dual-energy computed tomography in a multiparametric regression model for diagnosing lymph node metastases in pancreatic ductal adenocarcinoma

**DOI:** 10.1186/s40644-024-00687-7

**Published:** 2024-03-20

**Authors:** Sheng Li, Dongping Jiang, Linling Jiang, Shumei Yan, Lizhi Liu, Guangying Ruan, Xuhui Zhou, Shuiqing Zhuo

**Affiliations:** 1 Department of Radiology, Sun Yat-sen University Cancer Center, State Key Laboratory of Oncology in South China, Guangdong Provincial Clinical Research Center for Cancer, Guangzhou, 510060 China; 2 Department of pathology, Sun Yat-sen University Cancer Center, State Key Laboratory of Oncology in South China, Guangdong Provincial Clinical Research Center for Cancer, Guangzhou, 510060 China; 3 Department of Radiology, the Eighth Affiliated Hospital, Sun Yat-sen University, Shenzhen, 518036 China

**Keywords:** Dual-energy, Computed tomography, Pancreatic ductal adenocarcinoma, Lymph node metastases, Iodine density

## Abstract

**Objective:**

To investigate the diagnostic value of dual-energy computed tomography (DECT) quantitative parameters in the identification of regional lymph node metastasis in pancreatic ductal adenocarcinoma (PDAC).

**Methods:**

This retrospective diagnostic study assessed 145 patients with pathologically confirmed pancreatic ductal adenocarcinoma from August 2016–October 2020. Quantitative parameters for targeted lymph nodes were measured using DECT, and all parameters were compared between benign and metastatic lymph nodes to determine their diagnostic value. A logistic regression model was constructed; the receiver operator characteristics curve was plotted; the area under the curve (AUC) was calculated to evaluate the diagnostic efficacy of each energy DECT parameter; and the DeLong test was used to compare AUC differences. Model evaluation was used for correlation analysis of each DECT parameter.

**Results:**

Statistical differences in benign and metastatic lymph nodes were found for several parameters. Venous phase iodine density had the highest diagnostic efficacy as a single parameter, with AUC 0.949 [95% confidence interval (CI):0.915–0.972, threshold: 3.95], sensitivity 79.80%, specificity 96.00%, and accuracy 87.44%. Regression models with multiple parameters had the highest diagnostic efficacy, with AUC 0.992 (95% CI: 0.967–0.999), sensitivity 95.96%, specificity 96%, and accuracy 94.97%, which was higher than that for a single DECT parameter, and the difference was statistically significant.

**Conclusion:**

Among all DECT parameters for regional lymph node metastasis in PDAC, venous phase iodine density has the highest diagnostic efficacy as a single parameter, which is convenient for use in clinical settings, whereas a multiparametric regression model has higher diagnostic value compared with the single-parameter model.

**Supplementary Information:**

The online version contains supplementary material available at 10.1186/s40644-024-00687-7.

## Key Points

• In order to ascertain the presence of regional lymph node metastasis in pancreatic ductal adenocarcinoma, a regression diagnostic model was constructed utilizing various dual-energy computed tomography parameters.

• The dual-energy CT multi-parameter regression model demonstrates a notable diagnostic efficacy in detecting lymph node metastasis of PDCA, thereby providing valuable assistance in clinical decision-making.

**Supplementary Information:**

The online version contains supplementary material available at 10.1186/s40644-024-00687-7.

## Introduction

Pancreatic ductal adenocarcinoma (PDAC) is a common gastrointestinal tumor that is highly malignant. Because most patients are diagnosed at locally advanced intermediate or late stages, their prognosis is poor [1, 2]. With frequent recurrences and metastases, lymph node metastasis (LNM) is a significant prognostic factor that affects the patient’s outcome [1, 3–5].

Currently, routine imaging techniques cannot accurately predict preoperative nodal status [6]. Abdominal ultrasound examination does not typically provide a definitive diagnosis for abdominal LNM because of the presence of intestinal gas. Positron emission tomography/computed tomography (PET-CT) studies are expensive and not widely available [7, 8], with sensitivity: 71.2%-81.8%, positive predictive value: 68.1%, negative predictive value:79.1% [7, 9, 10]. Magnetic resonance imaging (MRI) offers excellent soft tissue resolution; however, its spatial resolution is lower than that of CT, making it difficult to evaluate LNM [11–13]. Conventional CT relies on different tissue densities to distinguish different lesions [14, 15], whereas dual-energy CT (DECT) can distinguish between lesions with the same density using multiple parameters. Numerous tumor can be correctly classified via nodal staging using quantitative parameters from CT data. Most studies have established regression models with good diagnostic value [16–19].

However, to date, it is not possible to clearly diagnose metastatic from non metastatic lymph nodes in PDAC. Furthermore, the diagnostic values of the DECT parameters for LNM in PDAC have not been compared in detail. The present study aimed to identify a DECT parameter with the highest diagnostic efficiency that is suitable for use in the clinical setting. Multiparametric DECT was used to diagnose LNM in PDAC, establish a diagnostic model, and compare the receiver operator characteristic curve (ROC) of each DECT parameter. These data allowed us to identify the single DECT parameter with the highest diagnostic efficiency, which is convenient for clinical use.

## Materials and methods

### Study population

**Fig. 1:**
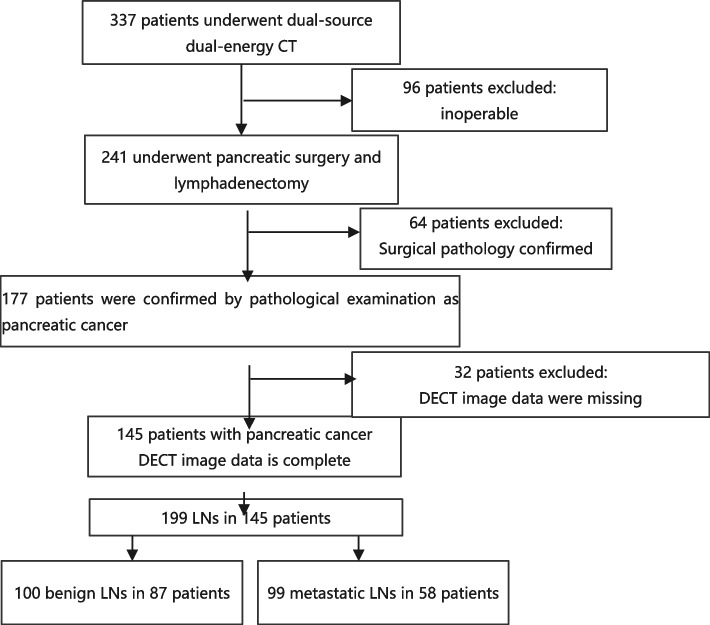
Flowchart of all patients

This was a single-institution, retrospective, diagnostic, descriptive study. Cumulative data were collected from August 2016 to October 2020 at Sun Yat-sen University Cancer Center, Guangzhou, China, for 953 patients with PDAC who received CT scan or MR scan. The regional lymph nodes were matched with the pathological results, and the patients were divided into two groups: a metastatic lymph node group (*n* = 99) and a benign lymph node group (*n* = 100). Based on the preoperative venous phase CT images, the critical inclusion criteria were: 1) pathologically confirmed PDAC and LNM and 2) DECT scanning within 1 month before surgical resection. Exclusion criteria were: 1) history of any systemic therapy before surgical resection and 2) missing preoperative clinical and image data. The complete patient enrollment process is shown in Fig. 1. This study was approved by the institutional ethics committee of Sun Yat-sen University Cancer Center, Guangzhou, China (IRB number: B2019-012-01). Informed consent was waived based on the retrospective study design.

### Imaging technique

All patients were scanned using a dual-source CT scanner (SOMATOM Force, Siemens Healthcare) with the dual-energy mode. The acquisition parameters were detector collimation set at 192 × 0.6 mm, and 2) tube voltage and tube current set as follows: pitch 0.9, A/B tube current 250/125 mAs, and tube voltage 100 kV/Sn 150 kV. The CARE Dose 4D automatic exposure control system was turned on. The contrast agent iopromide 370 (370 mg/mL iodine, Bayer, Mississauga, Ontario, Canada) was intravenously injected through the antecubital vein by a power injector (XD 8003 Ulrich) with a 20-gauge needle. Contrast agent application was controlled using the bolus-tracking technique in the descending aorta (signal attenuation threshold, 100 HU). Data acquisition was initiated after the threshold was reached in the abdominal aorta, with a mean delay of 7 seconds. After 20 mL of saline solution was injected at flow rates of 4.0 mL/second, 1.5 mL/kg of contrast agent was used, followed by 40 mL of saline solution at flow rates of 4.0 mL/second. After initiation of contrast agent injection, multiphasic scanning was started with a 30- to 35-second delay for the arterial phase, a 60- to 65-second delay for the portal phase, and a 180-second delay for the delayed phase.

### Image postprocessing and measurement

All the CT images were transferred to an off-line workstation (Siemens syngo.via VB20 software). Venous phase images were selected to reconstruct virtual monoenergetic images. We developed and standardized ROI outlining criteria in our study. Manual outlining of the major part (about 60%) of the solid portion of the lymph node was performed. And we prevented drawing two small ROI to avoid potential low signal-noise rate. We also keep the ROI always in the solid portion of the LN to avoid partial volume effect. All measurements were performed twice on two consecutive maximal slices in the selected lymph nodes, and their average values were calculated. The slope of the spectral Hounsfield unit curve (in Hounsfield unit per kiloelectron-volt [HU per KV]) on virtual monochromatic imaging, defined as the difference between the CT value at 40 keV and that at 70 keV divided by the energy difference (30 keV), was calculated as follows:

$$\lambda HU=\left(HU \,40 \,keV-HU \,70 \,keV\right) / 30$$

**Fig. 2:**
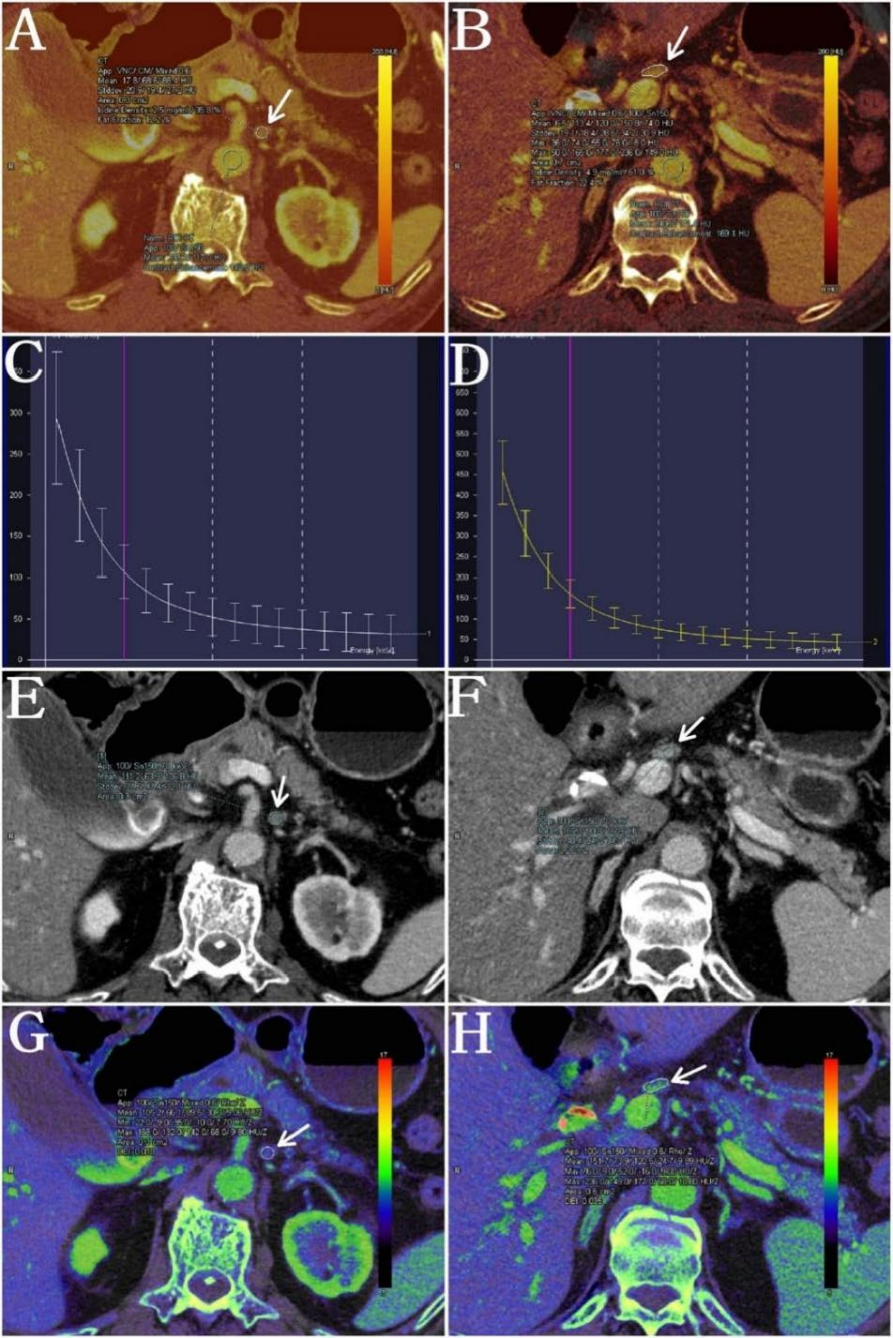
Measurement of dual-energy computed tomography (DECT) parameters in the same patients for different lymph nodes. The left column shows benign lymph nodes; the right column shows metastatic lymph nodes. Venous phase contrast-enhanced DECT images show the target lymph node with the following characteristics: iodine-based pseudo-colorized images for **A** a benign lymph node and **B** metastatic lymph node; representative effective atomic number images for **C** a benign lymph node and **D** a metastatic lymph node; representative venous phase CT images for **E** a benign lymph node and **F** a malignant lymph node; and graph of the spectral HU curves for **G** a benign lymph node, and **H** a malignant lymph node. CT, computed tomography; HU, Hounsfield unit

The HU 40 keV and HU 70 keV values represent the CT values measured on 40 keV and 70 keV images, respectively. Dual-energy parameters were measured by placing a region of interest such that it encompassed the solid portion of lymph nodes. The electron density (Rho), effective atomic number (Z), iodine concentration (IC), normalized iodine concentration (NIC), and the dual energy index (DEI) were obtained, and the differences were compared between the parameters for benign and metastatic lymph nodes. All measurements were performed twice on two consecutive maximal slices in the selected lymph nodes, and their average values were calculated. For cases in which the location was unclear, a radiologist-defined region of interest was determined based on pathological or surgical records (Fig. 2). The radiologists did not know the pathologic findings of the lymph nodes when they measured the energy spectrum parameters for diagnosis.

### Histopathologic evaluations

LN specimens were harvested by two pancreatic surgeon and the whole LN was fixed for HE staining with 5-um thin serial section. Surgical team members marked the resected specimen to dissect the lymph nodes. After the surgery, a microscope was used to show the regional LNM. Pathological results of included lymph nodes were used for gold standard by two pathologists with more than 6 years of experience. Pathology reports were obtained and reviewed for the patient sex and age and the tumor characteristics of location, size, differentiation status, growth pattern, and tumor–node–metastasis stage.

All regional lymph nodes in pancreatic ductal adenocarcinoma were divided into many levels in our hospital to ensure that the LNs selected on CT images corresponded to the pathological results, and surgeons usually strived to resect lymph node stations numbers 5, 6, 8a, 12, 13a, 13b, 14a, 14b, and so on (AJCC 8th edition). We collaborated with pathologists and surgeons. Before LNs dissection, target LNs were selected for spectral parameter measurements. We have adopted the following methods to ensure that the lymph nodes selected on abdominal CT images corresponded to the pathological results. We usually select lymph nodes in a specific station or region on preoperative CT. The size, shape, and three-dimensional location of the target LN in one node station were marked and recorded preoperatively on CT images by radiologists. We used coronal, axial, and sagittal CT images to help allocate LN. We measure its size on CT images and mark its relationship with blood vessels and nearby organ. During the surgery, the surgeon removes lymph node region by region and then labeled them. These targeted lymph nodes were put into some small clear bags with formalin solution separately, and the pathologist performed the diagnosis of the marked lymph node according to the lymph node station and records. It was shown that how we identified LNs in histopathology, as Supplemental Fig. 1. For lymph node in one known station, the following two methods have been further used in our study. First, according to the location (such as the subgroup, or nearby vessels), internal characteristics, shape and size of lymph nodes, doctors cooperate to match and correlate the size and morphological characteristics of the target lymph nodes under CT and microscope imaging. Second, if all lymph nodes in one region or station are malignant or benign, one node is selected for measurement- usually the largest one, for imaging-pathological correspondence.

### Statistical analysis

Statistical analyses were carried out using the statistical software SPSS, version 26.0 (SPSS) and MedCalc, version 19.3 (MedCalc Software). Continuous variables were presented as mean ± standard deviation (SD). Outliers were detected using box plots. The mean of the duplicate measurements was used in statistical analysis for all samples. The independent sample *t*-test or Mann–Whitney U test was used to compare continuous variables. The level of significance was set at *p* ≤ 0.05. The ROC curve, the areas under the ROC curve (AUC), 95% confidence interval (CI), sensitivity, specificity, Youden index, cutoff point, positive predictive value, and negative predictive value of single DECT parameter models and multiparameter diagnostic models were obtained using MedCalc, version 19.3, software, and these parameter models and multiparameter diagnostic models were also used to assess performance. The AUCs were compared using the Z test. A value of *p* < 0.05 was considered statistically significant.

The DeLong test was used to compare AUCs. A *p* value of <0.05 was considered statistically significant. Binary logistic regression analysis using a forward stepwise (conditional) method was performed. A regression model was established by combining multiple indicators with statistically significant (*p* values < 0.05), and variables with *p* values > 0.1 in univariate logistic regression models were excluded from the multivariable logistic regression models. This approach was based on research by Hosmer and Lemeshow (1989) on a goodness-of-fit test for the model, with a *p* value > 0.05 considered a good fit.

All diagnostic indicators were used to analyze the difference of the diagnostic value between benign and metastatic lymph nodes. The diagnostic efficacy of IC, predictive probability, and other indicators for lymph nodes < 5 mm and > 5 mm were compared, and the diagnostic efficacy of these indicators was compared for benign and metastatic lymph nodes.

## Results

A total of 145 patients, 60 women and 85 men aged 30–81 years (mean age 59.17 ± 9.94 years), met the study inclusion criteria (Table 1). Of these, 58 had LNM and 87 did not, and the shortest axis was measured for 99 metastatic and 100 benign lymph nodes, respectively. The short diameter of lymph nodes was 0.22–1.45 cm; this value was <5 mm in 52.3% and >5 mm in 47.7% of the lymph nodes.

**Table 1:** Baseline characteristics of the patients

Variable	Benign Lymph Nodes (*n*=100) in 87 patients	Metastatic Lymph Nodes (*n*=99) in 58 patients	*P*-value
Demographics			
Age(y), mean±SD	59.59±9.961	58.55±9.963	0.541
Gender			0.5
Female	38(43.68)	22(37.93)	
Male	49(56.32)	36(62.07)	
Histologic grade			0.066
Well differentiated	2(2.3)	1(1.7)	
Well to Moderately differentiated	5	0(0)	
Moderately to Poorly differentiated	38(43.68)	32(55.17)	
Moderately differentiated	33(37.93)	19(32.76)	
Poorly differentiated	4(4.60)	4(6.9)	
Other	5(5.71)	2(3.45)	
Lymph node size(cm), mean±SD	0.434±0.116	0.625±0.198	<0.0001
T stage			<0.0001
cTa-cT2	62(71.26)	17(29.31)	
cT3-cT4	25(28.74)	41(70.69)	
Metastasis			<0.0001
cM1	8(9.2)	1(1.7)	
cM0	79(90.8)	57(98.3)	

Data are number of patients; data in parentheses are percentage unless otherwise indicated

*Abbreviation*: *LNM* lymph node metastases *CT* computed tomography

**Fig. 3:**
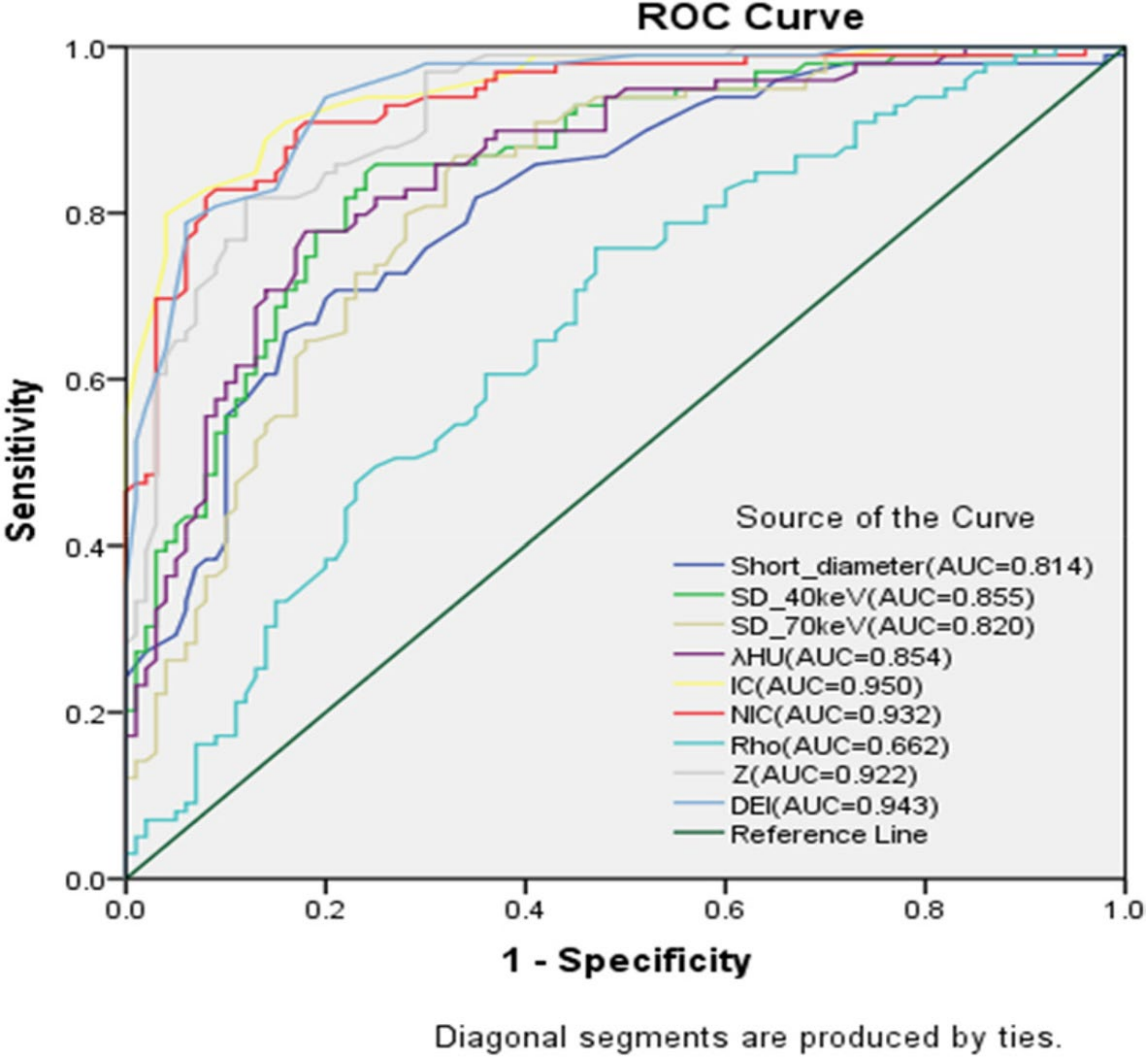
Comparison of receiver operating characteristic curves between different quantitative dual-energy computed tomography parameters for predicting lymph node metastasis in pancreatic ductal adenocarcinoma

**Fig. 4:**
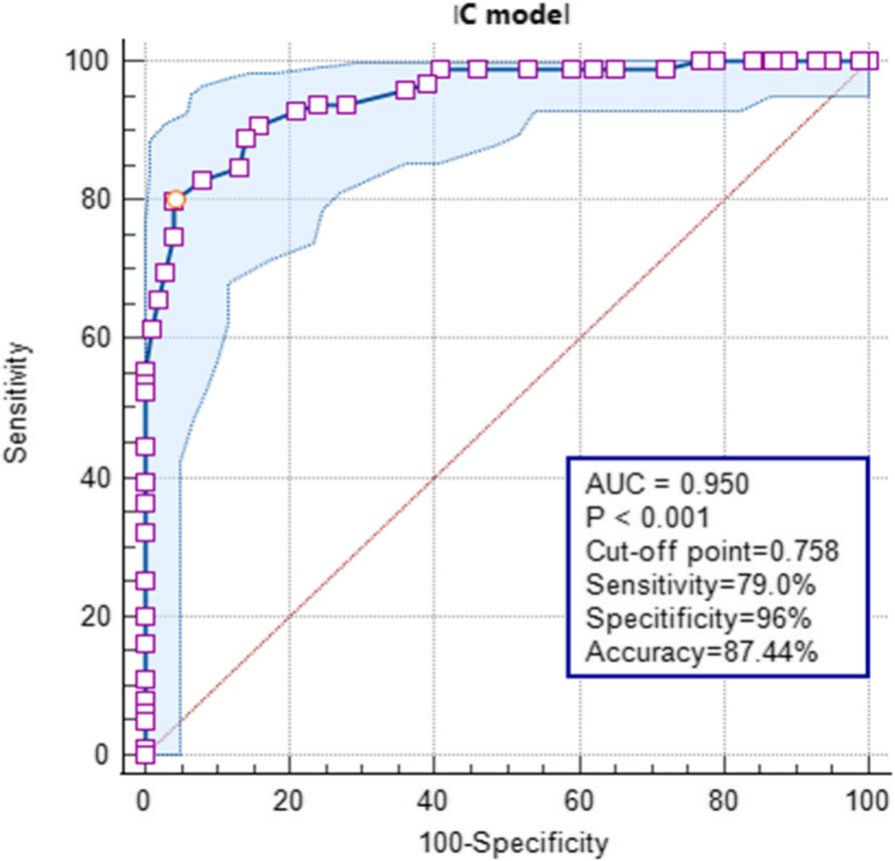
Receiver operating characteristic curve of quantitative dual-energy computed tomography parameters in the iodine concentration model

The comparison results of multiple parameters in the different lymph node groups are listed in Table 2. The p value of multiple DECT parameters was <0.05 for the benign and malignant lymph nodes, which is statistically significant. Figure 3 shows the comparison of the ROC curve between different quantitative DECT parameters for predicting LNM in PDAC. All single DECT parameter models have a defined significance for differentiating benign and metastatic lymph nodes in PDAC (Table 3). Among these, the IC model has the highest diagnostic efficiency with an AUC of 0.95 (95% CI, 0.915–0.972), diagnostic accuracy of 87.44% (174 of 199), sensitivity of 79.8%, and specificity of 96.0% (Table 4, Fig. 4).

**Table 2:** Quantitative Dual-Energy Computed Tomography Parameters to Discriminate Between Benign and Metastatic Lymph Nodes

Parameter	Benign Lymph Nodes (*n*=100)	Metastatic Lymph Nodes (*n*=99)	T	*P*-value
Short diameter	0.4334±0.11567	0.6247±0.19808	-8.311	0.000
SD-40 keV	64.292±13.9639	88.880±20.6417	-9.836	0.000
SD-70 keV	28.321±6.1689	36.707±7.7729	-8.434	0.000
Venous phase IC	2.814±0.7105	4.583±0.7779	-16.751	0.000
Venous phase NIC (%)	39.258±10.6296	65.027±14.4810	-14.320	0.000
Venous phase Rho	26.272±10.9276	32.287±9.5861	-4.126	0.000
Venous phase Z	8.9275±0.30117	9.5190±0.27827	-14.312	0.000
Venous phase DEI	0.02072±0.005614	0.03428±0.006078	-16.298	0.000
Venous phase λHU	5.4585±1.28651	7.35818±1.31711	-10.554	0.000

*DEI* dual energy index *IC* iodine concentration *NIC* normalized iodine concentration, *Z* effective atomic number

^*^Quantitative variables are presented as mean ± standard deviation

**Table 3:** Diagnostic Performance of Multiple Dual-Energy Computed Tomography Parameters in Diagnosing Lymph Nodes

Parameters	Short diameter	Venous phase λHU	SD-40 keV	SD-70 keV	Venous phase IC	Venous phase NIC	Venous phase Rho	Venous phase Z	Venous phase DEI	Multivariable logistic regression Model
No. of true positive	69	78	81	77	88	83	65	81	82	94
No. of false positive	20	21	24	23	14	15	43	15	15	5
No. of true negative	80	77	75	69	86	85	57	85	85	95
No. of false negative	30	23	19	30	11	16	34	18	17	5
Threshold	0.52	6.5483	70.80	20.95	3.95	54	26.85	9.26	0.02	0.66

**Table 4:** Prediction of Lymph Node Status Results and Performance Comparison of Different Quantitative Dual-Energy Computed Tomography Parameters

Parameters	AUC	cut-off point	95%CI	Threshold	Sensitivity (%)	Specificity (%)	PPV(%)	NPV (%)	Accuracy (%)
Short diameter	0.8140	0.4971	0.751~0.870	0.52	70.71	79.00	69.70	80.00	74.87
SD-40 keV	0.8550	0.6068	0.798~0.902	70.80	85.86	75.00	75.76	81.00	78.39
SD-70 keV	0.8200	0.5387	0.756~0.874	29.85	86.90	67.00	69.70	77.00	73.37
Venous phase IC	0.9500	0.7580	0.915~0.972	3.95	79.80	96.00	88.89	86.00	87.44
Venous phase NIC (%)	0.9320	0.7383	0.888~0.961	54.00	82.83	91.00	83.84	85.00	84.42
Venous phase Rho	0.6620	0.2876	0.584~0.741	26.85	75.76	53.00	65.66	57.00	61.31
Venous phase Z	0.9220	0.6982	0.881~0.952	9.26	81.82	88.00	81.82	85.00	83.42
Venous phase DEI	0.9430	0.7394	0.905~0.966	0.02	93.94	80.00	82.83	85.00	83.92
Venous phase λHU	0.8540	0.5978	0.794~0.902	6.5483	77.78	82.00	78.79	77.00	77.89
Multivariable logistic regression Model	0.9910	0.9196	0.975~0.997	0.66	95.96	96.00	94.95	95.00	94.97

^*^AUC area under the receiver operating characteristic curve, *DEI* dual energy index, *NPV* negative predictive value, *PPV* positive predictive value

According to the results of binary logistic regression analysis, among all DECT parameters, the short diameter, SD_70 keV, NIC, Rho, and dual energy index (DEI) were included in the regression equation based on a value of *p* < 0.05 (Supplemental Table 1), whereas SD_40 keV, λ HU, IC, and Z were excluded from the regression equation because of a value of p > 0.05. The multivariable logistic regression model was built with DECT parameters for predicting LNM using the following equation (threshold: 0.66):

Multivariable logistic regression model: y = 6.7 × Short_diameter + 0.148 × venous phase SD_70 keV + 0.142 × venous phase NIC + 0.095 × venous phase Rho +339.719 × venous phase DEI –27.838.

**Fig. 5:**
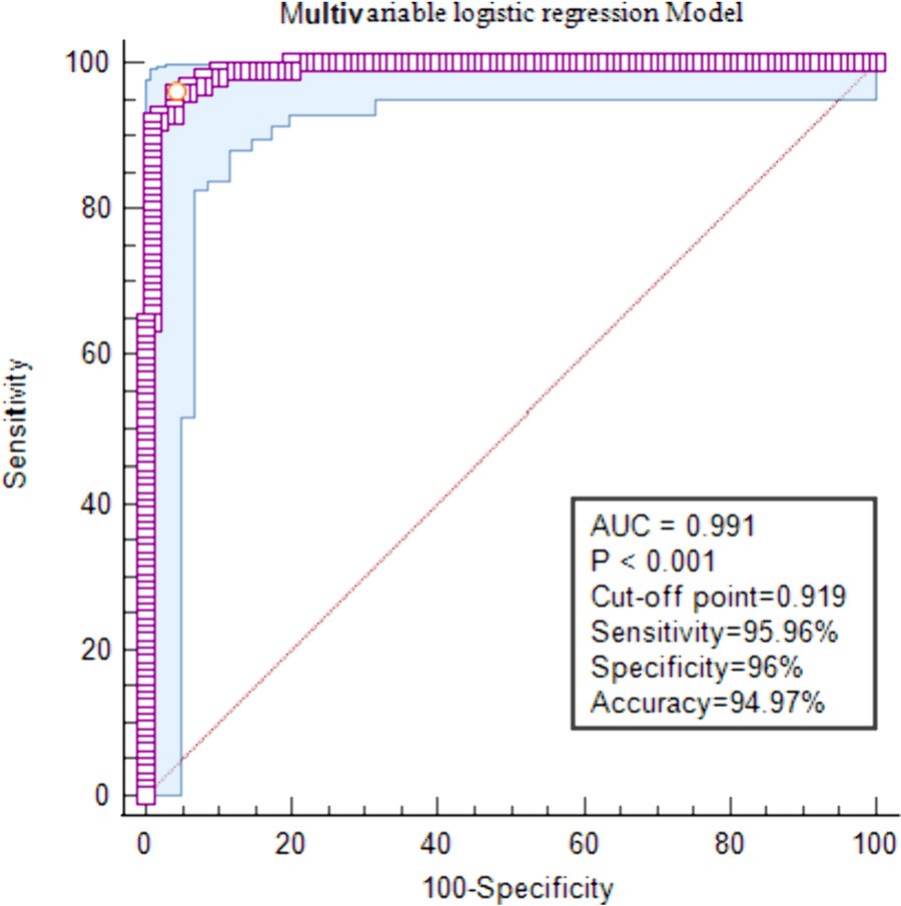
Receiver operating characteristic curve of the quantitative dual-energy computed tomography parameters model. The best threshold value for the predictive probability of binary logistic regression models of multiple parameters in differentiating between benign and metastatic lymph nodes was 0.66 cm. Area under the curve (AUC) was 0.991 (95% confidence interval, 0.975–0.997). The parameter model showed good discrimination

For this multivariable logistic regression model, AUC was 0.991 (95% CI, 0.975–0.997), diagnostic accuracy was 94.97% (189 of 199), sensitivity was 95.96%, and specificity was 96.0% (Table 4, Fig. 5). We have performed subgroup analysis (Supplemental table 2) and we did not find any difference between LNs with typical feature and those without in terms of iodine density.

**Fig. 6:**
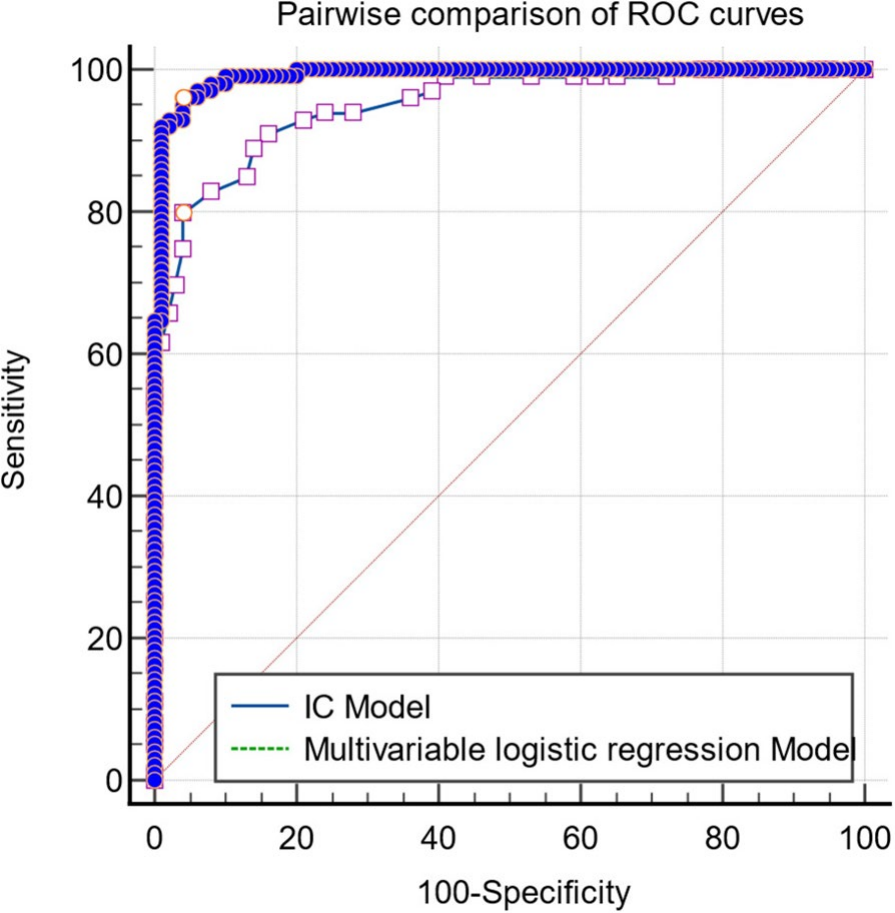
Pairwise comparison of receiver operating characteristic curves using the DeLong test. Result showed the difference between areas of ROC curves 0.0414 (95% confidence interval, 0.0177–0.0651) and a Z statistic of 3.424 with *p*= 0.0006, which is statistically significant

Pairwise comparison of ROC curves of the multivariable logistic regression models and IC model was performed using the DeLong test. Result showed a difference between areas of ROC curves 0.0414 (95% CI, 0.0177–0.0651; *p* = 0.0006 < 0.05), which is statistically significant (Fig. 6).

## Discussion

### The diagnostic efficiency of spectral parameters

In this study, DECT data in the venous phase were used to identify a number of quantitative parameters after postprocessing. The CT value of the 40 keV and 70 keV images were obtained and calculated to the slope of the energy spectrum curve from 40 keV to 70 keV as follows: λ HU = (HU 40 keV − HU 70 keV)/30 keV.

The DEI, Rho, Z, IC, and NIC were measured, and the following results were obtained. Multiple spectral quantitative parameters were used to differentiate between benign and malignant regional lymph nodes in PDAC. The results demonstrated that the multiparameter regression model had higher diagnostic value than any single spectral parameter in the differential diagnosis of LNM. By comparing the ROC curve of multiple DECT parameters, IC had the highest diagnostic efficiency for LNM in PDAC, with a remarkably high AUC of 94.9%, sensitivity of 79.8%, and specificity of 96%. These studies showed that NIC was not superior to IC in the diagnosis of LNM. Comparison of the ROC curve of IC and NIC revealed an AUC difference of 0.017, Z of 1.051, and *p* = 0.2932, which was much higher than *p* < 0.05, there was no statistically significant difference.

IC was strongly correlated with NIC, DEI, and Z, and compared with the other five study parameters, IC had better diagnostic performance. Therefore, for LNM in PDAC, it is more important to measure IC than other spectral parameters.

### ROC curve comparison

In terms of model quality evaluation, ROC curve comparison was performed and a precision recall curve was drawn, which indicated that the multiparameter energy spectrum model and IC had the highest diagnostic efficiency. To facilitate its clinical application, IC in the venous phase can be used to determine whether lymph nodes are metastatic, and most metastatic lymph nodes can be identified without any complicated calculations, which is more convenient than parametric models. However, the multiparameter regression model may be more useful for some lymph nodes (even rare) with IC values that are not significantly different from the threshold value.

### Traditional judgment criteria for LN metastasis

The accuracy of ultrasound and magnetic resonance imaging in the diagnosis of PDAC is insufficient. In addition, it is inaccurate to judge LNM based on the short diameter [13].The present study showed that >50% of benign lymph nodes had a short diameter of >4 mm. Also used as judgment criteria are DWI, heterogeneous enhancement, necrotic foci in lymph nodes, and morphological features [12, 20]; however, for lymph nodes with short diameter of <10 mm, image resolution and slice thickness are more important for diagnosis. In the earlier phases of nodal invasion, there is no obvious change in the node size, CT values, and the involvement of the lymph node capsule. At this time, it is difficult to use short diameter or density to assist diagnosis. In particular, based on the greater slice thickness and lower spatial resolution of conventional magnetic resonance imaging, it is difficult to clearly identify the signal changes exhibited by smaller lymph nodes. More than 50% of metastatic foci first appear in the marginal sinus of the lymph node [21]. Furthermore, in the early stages of LNM, it is challenging to determine the density and morphological changes of the whole lymph node. However, because the internal composition of lymph nodes has changed, even for the same density tissue, the spectral parameters may still reflect the difference of tissue composition and can be distinguished.

Different substances have different DEIs. This index can currently be used to distinguish more stable substances. Moreover, when the contrast agent is present, the DEI of the tissue increases in proportion to the concentration of the contrast agent.

Different substances usually have different dual energy index, and the dual energy index may increase as the concentration of the contrast agent in vivo increases; since the in vivo contrast agent is consistently variable for different tissues and times, the dual energy index may be variable. The poor diagnostic efficacy of DEI in the venous phase in this study may be attributed to these reasons; also, the venous phase scanning time varies in different hospitals.

### Radiology-pathology correspondence for LN

To ensure that the lymph nodes selected on CT images correspond with the pathological results, the following three methods have been used [22]. First, the imaging and pathological matching of lymph nodes can be performed according to the location, internal characteristics, shape and size of lymph nodes. The disadvantage of this correspondence method is that it is time-consuming and depends on the accuracy of lymph node labeling and grouping during surgical resection. Second, if all regional lymph nodes are malignant or benign, one node is selected for measurement. The disadvantage of this matching method is that if any of the lymph nodes are misdiagnosed or missed during pathological examination, it may lead to the erroneous imaging–pathological comparison. Third, for prospective studies, it is necessary for surgeons and pathologists to coordinate while marking on CT images preoperatively and perform pathological examination after marking to enable conducting a lymph node image–pathology correspondence study. This method of lymph node correspondence has the highest efficiency and is conducive to accurate correspondence; however, it is relatively difficult to implement.

In the present study, two adjacent maximum CT images of the same lymph node were measured, and the mean value and the specific location of the measurement were recorded to ensure the repeatability of the measurement. Using SPSS software to detect outliers, only <1% of the values were found to be changed due to human error. Postoperative recurrence of pancreatic cancer is often manifested as regional LNM. With timely and accurate diagnosis, it can be removed before the metastatic lymph node has progressed, which can reduce the severe pain caused by the invasion of the retroperitoneal nerve plexus and the resulting decline in the quality of life.

## Limitation and conclusion

The limitation of this study is that it cannot achieve a one-to-one correspondence between imaging and pathology of lymph nodes owing to its retrospective nature. The lack of external verification affected the statistical power to a certain extent. The morphological characteristics of lymph nodes were not determined, and the authors believe that in terms of lymph node morphology, lymph nodes with typical metastatic signs can usually be correctly diagnosed without spectral parameters.

In conclusion, the multiparameter spectral model had the highest diagnostic efficiency for LNM in PDAC, whether the lymph nodes were <5 mm or >5 mm. Venous phase iodine density has the highest diagnostic efficiency among single spectral parameters, and it can be used to accurately diagnose most regional lymph nodes in PDAC and is suitable for clinical application.

### Supplementary Information

**Additional file 1: Supplemental Figure 1. **How LNs were identified in histopathology A: lymph nodes removed in different stations after pancreatic cancer surgery. B: A single lymph node resected with fat, with long diameter about 25mm. C. A metastatic lymph node from pancreatic cancer under microscopy (H&E stain; original magnification×40). D. The same LN(H&E stain; original magnification×400). **Supplemental Table 1. **The DECT parameters were included in the regression equation. **Supplemental Table 2. **Subgroup analysis about difference between LNs with typical feature and those without in terms of iodine density.

## Abbreviations

AUC: The area under the curve
CT: Computed tomography
CI: Confidence interval
DECT: Dual-energy computed tomography
DEI: Dual energy index
HU: Hounsfield unit
IC: Iodine concentration
LNM: Lymph node metastasis
MRI: Magnetic resonance imaging
NIC: Normalized iodine concentration
PDAC: Pancreatic ductal adenocarcinoma
PET-CT: Positron emission tomography/computed tomography
ROC: The receiver operator characteristic curve
Rho: The electron density
SD: Standard deviation
Z: Effective atomic number
