# Supplementary material for: Dual-energy computed tomography in a multiparametric regression model for diagnosing lymph node metastases in pancreatic ductal adenocarcinoma
Source: Cancer Imaging. 2024 Mar 20;24:38. doi: 10.1186/s40644-024-00687-7 (PMC10953218; doi:10.1186/s40644-024-00687-7)
Supplement: Supplementary file 1 — Additional file 1: Supplemental Figure 1. How LNs were identified in histopathology A: lymph nodes removed in different stations after pancreatic cancer surgery. B: A single lymph node resected with fat, with long diameter about 25mm. C. A metastatic lymph node from pancreatic cancer under microscopy (H&E stain; original magnification×40). D. The same LN(H&E stain; original magnification×400). Supplemental Table 1. The DECT parameters were included in the regression equation. Supplemental Table 2. Subgroup analysis about difference between LNs with typical feature and those without in terms of iodine density. [file 40644_2024_687_MOESM1_ESM.docx]

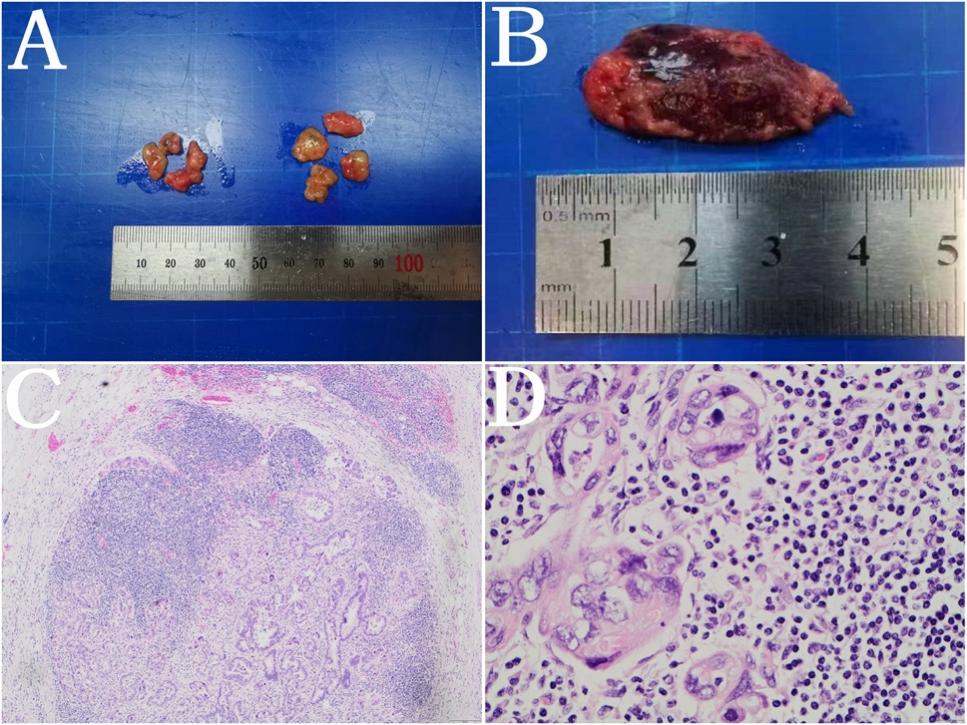


**Supplemental Figure 1 How LNs were identified in histopathology A: lymph nodes removed in different stations after pancreatic cancer surgery. B: A single lymph node resected with fat, with long diameter about 25mm. C. A metastatic lymph node from pancreatic cancer under microscopy (H&E stain; original magnification×40). D. The same LN(H&E stain; original magnification×400)**

**Supplemental Table 1.** The DECT parameters were included in the regression equation

| Variable | **Coefficient** | **Std. Error** | **Wald** | **P** | **Odds ratio** | **95% CI** |
| --- | --- | --- | --- | --- | --- | --- |
| Short_diameter | 6.79864 | 2.6709 | 6.4793 | 0.0109 | 896.6287 | 4.7763 to 168318.5949 |
| SD_70keV | 0.14752 | 0.058378 | 6.386 | 0.0115 | 1.159 | 1.0337 to 1.2995 |
| NIC | 0.14161 | 0.034871 | 16.4905 | <0.0001 | 1.1521 | 1.0760 to 1.2336 |
| Rho | 0.095195 | 0.047024 | 4.0981 | 0.0429 | 1.0999 | 1.0030 to 1.2061 |
| DEI | 339.71867 | 89.56203 | 14.3877 | 0.0001 | 3.45E+147 | 200.079E+69 to 59.5226E+222 |
| Constant | -27.83816 | 5.10191 | 29.7725 | <0.0001 |  |  |

* According to the results of binary logistic regression analysis, among all DECT parameters, the short diameter, SD_70 keV, NIC, Rho, and DEI were included in the regression equation

**Supplemental Table 2** Subgroup analysis about difference between LNs with typical feature and those without in terms of iodine density.

| typical feature | Test Result Variable(s) | Area | Std. Error^b^ | Asymptotic Sig.^c^ | Asymptotic 95% Confidence Interval | |  |
| --- | --- | --- | --- | --- | --- | --- | --- |
|  |  |  |  |  | Lower Bound | Upper Bound |  |
| 0 | λ HU | .888 | .029 | .000 | .832 | .945 |  |
|  | IC | .983 | .009 | .000 | .966 | 1.000 |  |
|  | NIC | .958 | .018 | .000 | .922 | .994 |  |
|  | Rho | .539 | .058 | .510 | .426 | .652 |  |
|  | Z | .937 | .021 | .000 | .895 | .978 |  |
|  | DEI | .950 | .024 | .000 | .902 | .998 |  |
| 1 | λ HU | .836 | .066 | .007 | .706 | .966 |  |
|  | IC | .956 | .039 | .000 | .880 | 1.000 |  |
|  | NIC | .891 | .083 | .002 | .727 | 1.000 |  |
|  | Rho | .684 | .138 | .137 | .413 | .955 |  |
|  | Z | .930 | .058 | .001 | .817 | 1.000 |  |
|  | DEI | .954 | .036 | .000 | .883 | 1.000 |  |
| a. For split file typical feature = 0, the test result variable(s): λ HU, IC, NIC, Rho, Z, DEI has at least one tie between the positive actual state group and the negative actual state group. Statistics may be biased. | | | | | | | |
| b. Under the nonparametric assumption | | | | | | | |
| c. Null hypothesis: true area = 0.5 | | | | | | | |
| d. For split file typical feature = 1, the test result variable(s): IC, DEI has at least one tie between the positive actual state group and the negative actual state group. Statistics may be biased. | | | | | | | |
